# Quiet Doubts: Exploring the Relationship between Introversion, Demographic Characteristics, and Imposter Phenomenon among Canadian Medical Students, Residents and Practicing Physicians

**DOI:** 10.5334/pme.2917

**Published:** 2026-08-25

**Authors:** Meghan M. McConnell, Neera R. Jain, Lindsay Cowley, Nancy L. Dudek, Victoria Luong, Timothy J. Wood, Kori A. LaDonna

**Affiliations:** 1 Department of Innovation in Medical Education, University of Ottawa in Ottawa, Canada; 2 Centre for Medical and Health Sciences Education, School of Medicine, Waipapa Taumata Rau, University of Auckland, in Auckland, New Zealand; 3 Ottawa Hospital Research Institute in Ottawa, Canada; 4 Department of Medicine, University of Ottawa in Ottawa, Canada; 5 Continuing Professional Development and Medical Education, Dalhousie University in Halifax, Canada; 6 Department of Innovation in Medical Education, University of Ottawa in Ottawa, Canada; 7 Department of Innovation in Medical Education and Department of Medicine, University of Ottawa in Ottawa, Canada

## Abstract

**Introduction:**

Medicine often privileges extroverted traits such as confidence, assertiveness, and decisiveness. This ‘extrovert ideal’ may disadvantage introverted trainees and physicians, increasing vulnerability to imposter phenomenon (i.e., persistent feelings of inadequacy and self-doubt). However, the relationship between introversion and imposter phenomenon remains poorly understood, particularly among marginalized demographic groups who may already be more susceptible to imposter feelings. This study explored associations between introversion, imposter phenomenon, and demographic factors among Canadian undergraduate medical students (UGME), postgraduate residents (PGME), and practicing physicians.

**Methods:**

We conducted a cross-sectional survey. Introversion was measured using the McCroskey Introversion Scale and imposter phenomenon using the Clance Imposter Phenomenon Scale (CIPS). Demographic variables included gender, disability status, race/ethnicity, sexuality, first-generation medical student status, and training level (UGME, PGME, and practicing physicians).

**Results:**

The final sample included 578 participants. Multiple linear regression showed that higher introversion scores were associated with higher CIPS scores (B = 0.68, 95% CI [0.52, 0.84], p < 0.001). Participants identifying as women (B = 6.18 [3.28, 8.80], p < 0.001) or disabled (B = 4.93 [1.91, 7.95], p = 0.001) also reported higher CIPS scores. UGME students reported higher CIPS scores than practicing physicians (B = 3.74 [0.46, 7.01], p = 0.03). No significant interactions were observed between introversion and demographic variables.

**Discussion:**

Higher introversion was associated with greater imposter phenomenon across all stages of medical training and practice. Women, individuals with disabilities, and UGME trainees also reported greater imposter phenomenon. These findings highlight the need for further research into factors underlying the relationship between introversion and imposter phenomenon in medicine, and how professional cultures may shape imposter phenomenon experiences across diverse groups of trainees and physicians.

## Introduction

Personality traits play a central role in how individuals navigate educational, professional, and personal contexts [1]. Among these traits, introversion and extroversion are perhaps the most widely recognized and intuitively understood [2]. Broadly speaking, extroverts tend to be more outgoing, assertive, and action-oriented, deriving energy from social situations, while introverts are more subdued, thought-oriented, and analytical, drawing energy from reflection and solitude [3]. While both dispositions have distinct strengths, Western cultures often privilege the gregarious, quick-thinking behaviours of extroverts to the detriment of introverts, a phenomenon referred to as the ‘extrovert ideal’ [2]. This cultural bias can have negative effects on individuals whose natural dispositions do not align with extrovert-oriented expectations, particularly in professional environments where such norms are deeply embedded.

Medicine may represent one such environment. Emerging evidence within medical education has described medical culture as implicitly rewarding extroverted traits while undervaluing the quieter, more reflective qualities of introverts [4,5,6,7,8,9]. For instance, clinical practice often rewards confidence and quick, decisive action [10,11,12], while training frequently emphasizes interactive, group-based learning [4,9]. Within such environments, introverts’ tendency to pause and reflect before responding can be misinterpreted as hesitation, indecisiveness, or even incompetence [4,13], pressuring them to ‘act extroverted’ to meet professional expectations [13,14]. Over time, this person-environment mismatch may foster feelings of self-doubt and inadequacy, undermining introverts’ sense of belonging and legitimacy.

Such experiences of self-doubt and inauthenticity align closely with the defining features of imposter phenomenon (also known as imposter syndrome), a form of psychological distress characterized by persistent feelings of inadequacy and an internalized fear of being exposed as a ‘fraud’, despite evidence of achievements [15]. Imposter feelings are widespread in high-achieving professions such as medicine, with a recent meta-analysis reporting that nearly 50% of medical trainees and physicians experience imposter phenomenon [16]. While imposter phenomenon was originally framed as a problem arising within the individual, rooted in a lack of confidence and low self-esteem [15,17,18], critics have challenged this framing, arguing that sociocultural factors play a critical role in shaping imposter feelings [19,20,21]. For example, research within higher education has shown that imposter phenomenon disproportionately affects members of marginalized communities, including women [22,23,24,25], individuals from racialized backgrounds [26,27,28], people with disabilities [29,30,31], first-generation students [32,33], and members of the lesbian, gay, bisexual, transgender, and queer (LGBTQ+) community [34]. These findings challenge the notion that imposter phenomenon is a purely individual experience, highlighting the role of context and social structure in fostering imposter feelings, particularly among those whose identities do not align with dominant sociocultural norms.

Introversion may represent one such case of person-environment misalignment, where extrovert-oriented preferences in high-achieving professions such as medicine may leave introverted individuals particularly vulnerable to imposter feelings. If imposter phenomenon is shaped, even partly, by the sociocultural norms and values embedded within medical training, then effective interventions require understanding it as a multifaceted professional challenge rather than solely an individual problem rooted in low self-esteem. Recent research on personality and imposter phenomenon provides preliminary evidence that introverts may be more susceptible to imposter feelings. For example, Kodweis and colleagues [35] investigated the relationship between imposter phenomenon and Myers-Briggs Type Indicator personality types among pharmacy students, and found that introverted learners were nearly twice as likely to experience high or severe levels of imposter phenomenon compared to their extroverted peers. Similarly, Sawant et al [36] explored the relationship between imposter phenomenon, self-esteem, and Big-5 personality types, and reported lower imposter phenomenon among more extroverted medical students. While these findings provide preliminary evidence that introverted healthcare trainees may be at an elevated risk for imposter feelings, neither study specifically examined introversion-extroversion across the medical education continuum. This understanding is imperative because, over time, a person-environment mismatch risks fostering feelings of self-doubt and inadequacy, undermining introverted trainees’ and practicing physicians’ sense of belonging and legitimacy.

Despite growing recognition of the challenges faced by introverted medical trainees and physicians, more research is needed to better understand the relationship between introversion and imposter phenomenon, particularly among individuals in medicine from marginalized communities who may already be more vulnerable to imposter feelings. To address this gap, we examined whether introversion was related to imposter phenomenon among a sample of Canadian undergraduate medical students, postgraduate trainees, and practicing physicians, while also exploring whether this relationship varied across demographic characteristics previously linked to elevated imposter phenomenon. Specifically, we aimed to address the following research question: What is the relationship between introversion, demographic characteristics (gender, race/ethnicity, sexuality, disability status, first-generation medical student status, and training level), and imposter phenomenon among Canadian medical trainees and physicians?

## Methods

### Study Design

We conducted a cross-sectional online survey July 2024 to May 2025 using SurveyMonkey (SurveyMonkey Inc., San Mateo, California). The data reported here were drawn from a larger national survey examining how introversion and extroversion shape educational and professional experiences across the medical education continuum (in preparation). Because the broader survey was designed to explore multiple related but conceptually distinct research questions, findings are reported in separate manuscripts to allow for a more conceptually coherent and interpretable analysis [37]. The current analysis focuses specifically on the relationship between introversion and imposter phenomenon. The study received ethics approval from the University of Ottawa Health Sciences and Sciences Research Ethics Board (#H-05-24-10159, approved July 17, 2024).

### Context and Participants

In Canada, medical training follows a structured continuum across 17 medical schools^1^ [38], with most medical students having completed a 4-year bachelor’s degree prior to entering medical school. Undergraduate medical education (UGME) spans 3–4 years, comprising pre-clinical education followed by clinical clerkship. Upon completing UGME, graduates apply to postgraduate medical education (PGME) residency programs, with training duration varying across clinical specialties, ranging from two to five or more years. Some PGME trainees pursue additional subspecialty training through fellowship programs, typically lasting one to two years. Upon completion of PGME and necessary licensure examinations, physicians are eligible to enter independent practice [39]. Our study recruited participants across all stages of this continuum, including current UGME medical students and PGME trainees (hereafter referred to collectively as ‘trainees’), as well as practicing physicians. We estimated the minimum required sample size using Green’s method (n > 50 + 8 m, where ‘m’ represents the number of variables) [40]. Based on our planned model, which included 9 predictor variables, we required a minimum of 122 participants to ensure adequate statistical power.

### Materials

#### Demographic questionnaire

We collected data on participants’ demographic characteristics, including their age, training level (UGME, PGME, or practicing physician), gender, sexuality, disability status, ethnicity, and first-generation medical student status. Gender and sexuality were collected using open-ended responses to allow participants to self-identify in their own terms rather than selecting from predefined categories [41]; for gender, participants were informed that “current gender may be different from sex assigned at birth”. Remaining variables were selected from drop-down menus with an “Other” option and open-text field. Drop-down response options for disability status were drawn from the Association of American Medical Colleges Medical School Questionnaire [42], and all other drop-down response options were taken from the Canadian Medical Association National Physician Health Survey [43].

#### Introversion

We measured introversion using the McCroskey Introversion Scale (MIS), a self-report measure designed to assess introversion without being conflated by communication apprehension behaviours commonly associated with social anxiety [44,45]. The MIS consists of 12 items measured with a 5-point Likert scale (1 = strongly disagree to 5 = strongly agree). Scores range from 12 to 60, with higher scores indicating higher introversion and lower scores indicating lower introversion (e.g., higher extroversion). Previous studies have reported high internal consistency of MIS scores across different cultures (Cronbach’s α = 0.80–0.90) [44,46,47,48,49]. The internal consistency of the MIS was a = 0.89 in the present study.

#### Imposter Phenomenon

We measured imposter phenomenon using the Clance Imposter Phenomenon Scale (CIPS), a 20-item scale rated on a 5-point Likert scale (1 = strongly disagree to 5 = strongly agree) [50]. Scores range from 20 to 100, with higher scores indicating greater imposter phenomenon. Previous studies have demonstrated high internal consistency of CIPS across multiple populations (α = 0.85–0.96) [51,52]. The internal consistency of the CIPS was a = 0.93 in the present study.

### Data Collection

Recruitment materials were distributed to communications and medical education departments across 17 Canadian medical schools, with requests for broad dissemination. At the UGME and PGME levels, materials were shared via research information boards, newsletters, and direct email distribution to trainees and faculty. At the PGME level, recruitment emails were also sent to program administrators for further distribution to residents and faculty. Additional recruitment occurred through social media (Twitter/X), the Canadian Federation of Medical Students listservs, and local medical education professional networks circulated by the study authors.

Recruitment materials included a description of the study, contact information for the research team, and a link to the online survey. Upon accessing the survey link, respondents were presented with a consent form, which had to be accepted before answering survey questions. Participants could withdraw at any time. As an incentive, individuals could enter a draw to win one of three $400 gift cards.

### Data Analysis

We used IBM SPSS 31 (IBM Corp, Armonk, NY) for all analyses and considered p-values ≤ 0.05 to be statistically significant. Descriptive statistics were used to summarize data, including measures of central tendency and dispersion for continuous variables and counts and percentages for categorical data. For analytical purposes, demographic categories with small cell sizes were combined: participants identifying as trans, nonbinary, agender, or genderqueer were grouped into a “Gender Diverse” category, and those identifying as gay, queer, lesbian, bisexual, pansexual, asexual, demisexual, or biromantic were aggregated into a “Sexual and Relationship Minority (SRM)” category. Disability status was dichotomized as “with” or “without” a disability; participants were classified as having a disability if they selected any listed option or provided a written response in the “Other” category. Drawing on prior literature on underrepresented ethnicities in Canada [53,54], participants who identified as Black/African American, Filipino, Hispanic/Latino, or Indigenous were categorized as underrepresented in medicine (URIM).

Multiple linear regression was used to examine the relationship between introversion, demographic characteristics, and imposter phenomenon, with mean CIPS scores as the outcome measure and MIS scores and demographic variables (gender, disability status, SRM status, URIM status, first-generation medical student status, and training level) as predictors. Respondents categorized as “gender diverse” (1%) were not incorporated due to small sample size. Consequently, all demographic variables were treated as dichotomous, with the exception of training level, which was dummy coded with practicing physicians as the reference group. To compare the relative strength of each predictor within the model, we reported standardized regression coefficients (β), which place all predictors on a common scale and allow direct comparison of their associations with CIPS scores regardless of their original units of measurement.

Given the exploratory nature of this analysis, all demographic variables were first entered into the model without interaction terms. To examine whether the relationship between introversion and imposter phenomenon varied across demographic characteristics, interaction effects were tested in separate regression models, which allowed us to determine whether the strength or direction of the relationship between MIS and CIPS varied across demographic variables. Specifically, interaction terms between MIS scores and each demographic variable were entered individually into models that also included the corresponding main effects. Prior to analysis, we confirmed that the data met the standard requirements for multiple linear regression, including independence of residuals, homoscedasticity, and absence of multicollinearity. Effect sizes were calculated using Cohen’s *f^2^*, with values of 0.02, 0.15, and 0.35 corresponding to thresholds for small, medium, and large effect sizes, respectively [55].

## Results

A total of 638 responses were collected from participants across ten Canadian provinces. Of these, two were removed for failure to provide consent and 45 were removed due to incomplete responses (defined as more than 80% of the survey items missing). During routine data quality review, we identified nine responses as likely bot submissions based on identical response patterns across survey items and identical survey completion timestamps; these were removed. Lastly, four participants completed the survey twice, and in these cases, only the first response was retained. The final sample consisted of 578 participants.

### Sample Characteristics

Demographic characteristics of participants, along with corresponding mean MIS and CIPS scores, are presented in Table 1. Most respondents were either UGME students (n = 235; 41%) or PGME trainees (n = 213; 37%), with practicing physicians representing 22% (n = 130) of the sample. Participants ranged from 21 to 77 years of age (mean (SD) = 32.4 (9.7)). Approximately 70% (n = 398) of participants identified as women, 17% (n = 92) as members of the SRM community, 23% (n = 127) were persons with a disability, 9% (n = 49) identified as being from an URIM ethnicity and 77% (n = 436) were the first-generation in their family to attend medical school.

**Table 1:** Demographic characteristics of study participants, as well as mean (standard deviation) McCroskey Introversion Scale (MIS) and Clance Imposter Phenomenon Score (CIPS) scores.


VARIABLE	N (%)	MIS	CIPS

Gender^2^			

Women	398 (69.9%)	31.6 (7.3)	65.1 (14.2)

Men	165 (29.0%)	32.6 (8.0)	59.2 (14.7)

Gender Diverse	6 (1.1%)	36.5 (5.7)	74.8 (9.6)

Sexual and relationship minority			

No	438 (82.6%)	31.9 (7.3)	63.2 (15.1)

Yes	92 (17.4%)	31.3 (8.3)	65.5 (12.8)

Race and Ethnicity			

non-URIM	483 (90.8%)	31.8 (7.5)	63.2 (14.8)

URIM	49 (9.2%)	31.5 (8.2)	65.9 (13.7)

Disability			

No	434 (77.4%)	32.0 (7.5)	62.1 (14.5)

Yes	127 (22.6%)	31.7 (7.4)	68.4 (14.0)

First-generation medical student			

No	128 (22.7%)	32.5 (7.1)	64.3 (15.8)

Yes	436 (77.3%)	31.8 (7.6)	63.2 (14.3)

Training Level			

UGME	235 (40.7%)	31.1 (7.2)	64.8 (13.5)

PGME	213 (36.9%)	31.7 (7.6)	64.1 (14.5)

Practicing physician	130 (22.5%)	33.8 (7.5)	60.37 (16.3)


URIM = under-represented in medicine; UGME = undergraduate medical education; PGME = postgraduate medical education. *Note*. Responses to demographic questions were optional; therefore, totals may not sum to 578.

### Relationship between Introversion, Demographic Variables, and Imposter Phenomenon

**Figure 1:**
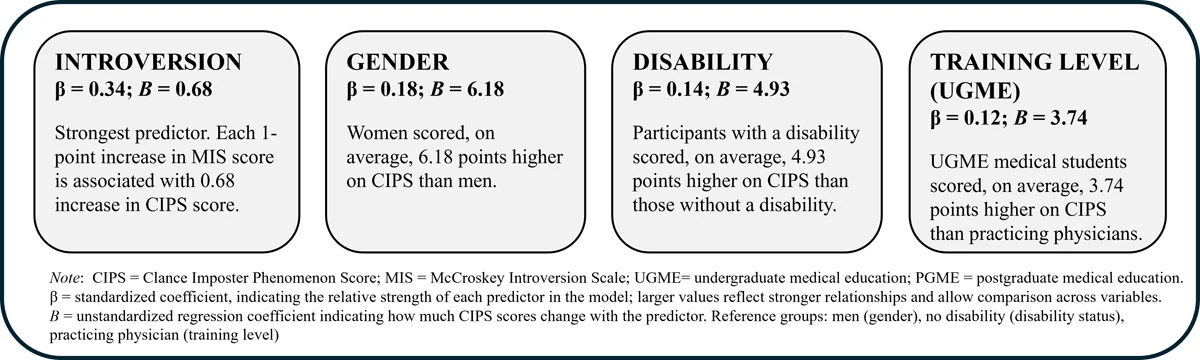
Significant predictors of Imposter Phenomenon (CIPS) from multiple linear regression. Each box displays the standardized (β) and unstandardized (*B*) regression coefficients, along with an interpretation of the association. Non-significant predictors (PGME training level, race/ethnicity, sexual and relationship minority status, and first-generation medical student status) are not shown.

The multiple linear regression model explained 18% of the variance in CIPS scores (Table 2; *F* = 12.3, p < 0.001, R^2^_adj_ = 0.18, Cohen’s *f*^2^ = 0.22). Four variables were significantly associated with CIPS scores (Figure 1). First, MIS scores were positively associated with CIPS scores, indicating that participants with higher levels of introversion reported greater levels of imposter phenomenon (B = 0.68, 95% CI [0.52, 0.84], β = 0.34, p < 0.001). Second, gender was significantly associated with CIPS scores: women reported higher CIPS scores than men (B = 6.18 [3.37, 8.99], β = 0.18, p < 0.001). Third, participants who reported having a disability had higher CIPS scores than those without a disability (B = 4.93 [1.91, 7.95], β = 0.14, p = 0.001). Lastly, training level was associated with CIPS scores. Compared to practicing physicians, UGME students reported higher CIPS scores (B = 3.74 [0.46, 7.01], β = 0.12; p = 0.03); however, CIPS scores did not differ between PGME trainees and practicing physicians (p = 0.09). Based on standardized regression coefficients, introversion showed the strongest independent association with CIPS (β = 0.34), roughly twice the magnitude of the next strongest predictor, gender (β = 0.18).

**Table 2:** Multiple linear regression coefficients for imposter phenomenon (CIPS).


PREDICTOR VARIABLE	UNSTANDARDIZED REGRESSION COEFFICIENT (B)		STANDARDIZED REGRESSION COEFFICIENT (β)

	B	95% CONFIDENCE INTERVAL	

Introversion (MIS)	0.68^***^	0.52–0.84	0.34

Gender (vs. Men)			

Women	6.18^***^	3.37–8.99	0.18

Sexual and relationship minority (vs. No)			

Yes	0.37	–3.04–3.79	0.01

Race and Ethnicity (vs. non-URIM)			

URIM	2.97	–1.35–7.28	0.06

Disability (vs. No)			

Yes	4.93^***^	1.91–7.95	0.14

First-generation medical student (vs. No)			

Yes	–0.28	–3.25–2.69	–0.01

Training Level (vs. Practicing Physician)			

UGME	3.74^*^	0.46–7.01	0.12

PGME	2.83	–0.44–6.10	0.09


*p ≤ 0.05, **p ≤ 0.01, ***p ≤ 0.001. CIPS = Clance Imposter Phenomenon Score; MIS = McCroskey Introversion Scale; URIM = under-represented in medicine; UGME = undergraduate medical education; PGME = postgraduate medical education. Unstandardized regression coefficient (*B*) indicates how much CIPS scores change with the predictor. Standardized regression coefficient (β) indicates the relative strength of each predictor in the model; larger values reflect stronger relationships and allow comparison across variables.

Subsequent linear regressions indicated that none of the interaction terms between MIS scores and demographic variables were statistically significant (all p > .10), suggesting that the relationship between MIS and CIPS did not differ significantly across demographic groups.

## Discussion

The present study found that higher levels of introversion were associated with greater imposter phenomenon among medical trainees and practicing physicians; this association remained consistent across demographic groups, suggesting that introverted trainees and physicians may be at elevated risk for imposter phenomenon across a range of demographics and career stages. Additionally, we found that women, individuals with disabilities, and UGME trainees reported higher imposter phenomenon than their peers.

The observed association between introversion and imposter phenomenon is unlikely to be attributable to a single factor. Rather, it likely reflects an interplay between individual dispositions and the broader sociocultural contexts in which those dispositions are expressed. At the individual level, several characteristics commonly associated with introversion may contribute to heightened imposter feelings. Relative to extroverts, introverts tend to report lower self-confidence, self-esteem, and sense of belonging [56,57,58,59], each of which has been linked to greater imposter phenomenon [60,61,62]. Introversion has been associated with greater sensitivity to negative social evaluation [63], which may be particularly salient in achievement-oriented, evaluative environments such as medicine. Finally, social support is a known protective factor against imposter phenomenon [64], yet introverts are less likely to seek out support during periods of stress [65].

Additionally, cultural norms, social roles, and systemic structures within achievement-oriented environments like medicine may amplify imposter feelings among introverts, whose natural dispositions do not align with dominant professional norms. Consistent with this view, several large-scale studies have shown that psychological outcomes are shaped in part by how well an individual’s personality matches the dominant norms of the broader sociocultural context [66,67]. This raises the question of whether introverts actually perceive themselves as imposters within medicine, or whether their imposter feelings are, at least in part, externally imposed. Recently, LaDonna and colleagues [68] differentiated between imposter phenomenon and the Intruder Paradox, a socially reinforced experience in which individuals who do not fit professional norms face externally imposed perceptions of incompetence. In other words, “while imposters think ‘I don’t belong’ conflicting with objective evidence that they do, intruders are told ‘you don’t belong’” [68] (p 1063). If introverted physicians and trainees are experiencing the Intruder Paradox rather than (or in addition to) imposter phenomenon, then interventions focused solely on building individual resilience or confidence may miss the mark. Addressing these dynamics meaningfully will require research that moves beyond identifying who experiences imposter phenomenon and towards examining modifiable features of clinical learning and practice environments that give rise to these experiences in the first place.

Our study also identified significant associations between imposter phenomenon, gender, disability status, and training level. We found that women reported greater imposter phenomenon than men and that UGME trainees reported greater imposter phenomenon than practicing physicians, both consistent with prior literature (e.g., for reviews, see [69,70]. However, research examining imposter phenomenon among persons with disabilities remains notably limited in medical education. Within higher education, emerging evidence suggests that neurodivergent learners (e.g., those with Attention-Deficit/Hyperactivity Disorder or autism), or those with chronic health conditions experience greater imposter phenomenon than their non-disabled peers [29,30,31]. The near absence of research on imposter phenomenon among medical trainees and professionals with disabilities represents a critical and understudied gap given established knowledge about ableism in medicine [71,72,73,74].

Our findings have practical implications for medical training and practice. Medical educators can play a meaningful role by normalizing introversion as a legitimate professional style and identifying educational and clinical practices that may unintentionally reinforce feelings of self-doubt, inadequacy, and not belonging among introverts. Creating environments where introverted ways of engaging are recognized and valued may help reduce conditions that contribute to imposter feelings in the first place.

### Limitations

Participation in the study was voluntary, which introduced the potential for selection bias; trainees and healthcare providers who identify more strongly as introverted or extroverted may have been more inclined to participate. Additionally, the absence of national data on key demographic characteristics among Canadian medical trainees and practicing physicians (e.g., disability status, ethnicity, sexuality) made it difficult to precisely calculate a meaningful response rate, limiting our ability to assess non-response bias or determine how representative our sample is of the broader population. Some differences between our sample and the broader population are worth noting. Women are overrepresented in our sample relative to the Canadian medical workforce more broadly, while practicing physicians were underrepresented relative to UGME and PGME trainees [75,76,77]. Readers should therefore interpret our findings with caution with respect to generalizability, particularly for subgroups that may be underrepresented in our sample. Second, the study relied on self-report measures, which may be subject to reporting bias or social desirability effects. While the CIPS has been validated across multiple populations and research contexts, there is comparatively limited psychometric evidence supporting the validity of the MIS, which may limit the precision and interpretability of introversion-related findings. Third, our cross-sectional design precludes conclusions about causality. It remains unclear whether introversion predisposes individuals to imposter feelings, or whether repeated experiences of imposter phenomenon reinforce introverted coping behaviors such as withdrawal or self-containment. Fourth, for analytical purposes, demographic variables were dichotomized, which limited our ability to capture within-group variability and may have reduced statistical power to detect interaction effects. Fifth, small sample sizes precluded inclusion of gender-diverse participants in our analyses, limiting our ability to examine interactions between gender diversity, personality and imposter phenomenon. Additionally, gender was assessed using an open-ended item which did not allow differentiation between cisgender and transgender identities. Lastly, sample size constraints precluded analyses that examined multiple demographic characteristics simultaneously, limiting our ability to explore how intersecting social statuses, in combination with personality, may shape imposter phenomenon.

## Conclusion

The present study demonstrated that introversion was independently associated with imposter phenomenon across the medical education continuum, alongside gender, disability status, and training level. These findings support emerging research that imposter phenomenon in medicine reflects both individual characteristics and the professional norms within which trainees and physicians work. More research is needed to understand the mechanisms underlying these associations, and to explore what features of clinical training and practice environments may contribute to imposter feelings across diverse groups of trainees and practicing physicians.

## Additional File

The additional file for this article can be found as follows:

10.5334/pme.2917.s1 Supplemental Materials. McCroskey Introversion Scale (MIS) and Clance Imposter Phenomenon Scale (CIPS), including scale items and scoring instructions.

## Footnotes

At the time of data collection (July 2024–May 2025), 17 medical schools were operating in Canada. After the conclusion of this study, an 18^th^ institution enrolled its inaugural class in summer 2025 and a 19^th^ will enroll its inaugural class in 2026.

Gender categories were derived from open-ended responses. Participants categorized as *women* identified using terms such as *cis-woman, woman, cis-female*, or *female*; participants categorized as *men* identified using terms such as *cis-man, man, cis-male*, or *male*. The *gender diverse* category included participants who identified as *trans, nonbinary, agender*, or *genderqueer*.
