# Supplementary material for: Quiet Doubts: Exploring the Relationship between Introversion, Demographic Characteristics, and Imposter Phenomenon among Canadian Medical Students, Residents and Practicing Physicians
Source: Perspect Med Educ. 2026 Aug 25;15(1):663–74. doi: 10.5334/pme.2917 (PMC13523804; doi:10.5334/pme.2917)
Supplement: Supplementary file 1 [file pme-15-1-2917-s1.pdf]

For the best experience, open this PDF portfolio in Acrobat X or Adobe Reader X, or later.

Get Adobe Reader Now!
